# Whole-genome sequence characterization of respiratory syncytial virus in the Johns Hopkins Health System during the 2024–2025 respiratory season

**DOI:** 10.1128/spectrum.02065-25

**Published:** 2025-10-07

**Authors:** Ting Xuan Zhuang, Amary Fall, Julie M. Norton, Omar Abdullah, David A. Villafuerte, Andrew Pekosz, Eili Klein, Heba H. Mostafa

**Affiliations:** 1 Department of Pathology, Division of Medical Microbiology, Johns Hopkins School of Medicine 1500 , Baltimore, Maryland, USA; 2 Department of Emergency Medicine, Johns Hopkins School of Medicine 1500 , Baltimore, Maryland, USA; 3 W. Harry Feinstone Department of Molecular Microbiology and Immunology, The Johns Hopkins Bloomberg School of Public Health, Baltimore, Maryland, USA; 4 Center for Disease Dynamics, Economics, and Policy 439584, Washington, DC, USA; Oklahoma State University College of Veterinary Medicine, Stillwater, Oklahoma, USA

**Keywords:** genomic surveillance, sequencing, respiratory syncytial virus, RSV

## Abstract

Respiratory syncytial virus (RSV) remains a leading cause of lower respiratory tract infections in young children, older adults, and immunocompromised individuals worldwide. In 2023, the first RSV vaccines and the widespread use of monoclonal antibodies were approved, underscoring the need for genomic surveillance to monitor their effectiveness and the emergence of antibody-resistant genotypes. This study details the whole-genome characterization of RSV strains circulating between September and December 2024. Whole RSV genome amplification was performed using overlapping amplicons, followed by Nanopore sequencing. Viral genomes were analyzed to determine subtypes, clade prevalence, and amino acid substitutions. Epidemiological and clinical data were collected to assess associations with specific viral variants. A total of 336 RSV-positive samples were collected for the study. RSV activity followed expected seasonal patterns, with a predominance of RSV-A (94.9%) and the regional evolution of clade A.D.1.6 (68.34%). Over half of the infections occurred in children aged 1–5 years (56.2%). Comorbidities, including immunosuppression, were significantly associated with severe clinical outcomes. Phylogenetic analyses revealed tight clustering and low intra-clade diversity. Our results highlight the ongoing genetic evolution of RSV-A following the coronavirus disease 2019 (COVID-19) pandemic. Amino acid substitutions were detected across surface and internal proteins, potentially affecting the effectiveness of vaccines, monoclonal antibodies, or antivirals. Predicted gains and losses of glycosylation sites may further influence antigenic presentation. These findings underscore the need for integrated RSV genomic and epidemiological surveillance.

**IMPORTANCE:**

With ongoing antiviral drug development, recent approvals of new vaccines, and the continued use of protective antibody therapies, it is crucial to monitor the genetic evolution of RSV. Here, we present a long-amplicon-based whole-genome sequencing protocol for RSV-A and RSV-B, enabling genomic surveillance and representing the first report of whole-genome analysis of RSV strains circulating in areas served by the Johns Hopkins Health System during the 2024–2025 respiratory viral season. Our findings demonstrate the value of whole-genome surveillance in identifying emerging clades and molecular variations, and highlight the continued genomic evolution of RSV-A in the post–COVID-19 era.

## INTRODUCTION

Respiratory syncytial virus (RSV) is a major cause of lower respiratory tract infections in children, elderly, and immunocompromised individuals, accounting for approximately 1 in every 50 childhood deaths globally (1). While ribavirin remains the only Food and Drug Administration-approved antiviral for RSV, its use is limited due to modest efficacy and toxicity concerns (2). However, several vaccines were recently approved (GSK, AREXVY, Pfizer, Abrysvo, Moderna, and mRESVIA) and monoclonal antibodies (nirsevimab, AstraZeneca/Sanofi, Beyfortus) to provide protection for infants, the elderly, and the immunocompromised (3, 4). A minimal number of mutations in the viral glycoproteins have been shown to be associated with host immune evasion (5). Understanding the genomic evolution of RSV and the changes that may reduce the effectiveness of recent preventive immunization approaches is essential.

RSV belongs to the *Pneumoviridae* family and carries a ~15.2 kb negative-sense RNA genome comprising 10 genes that encode for 11 proteins (6). RSV is classified into two major subgroups: RSV-A and RSV-B (7). The RSV surface attachment (G) protein mediates viral binding to host epithelial cells, while the fusion (F) protein facilitates membrane fusion and viral entry (8, 9). These glycoproteins are major targets of neutralizing antibodies and exhibit substantial antigenic variability (10). Based on the genetic variability in the G gene, RSV-A and RSV-B have been divided into different genotypes (11, 12). However, more recently, a phylogenetic classification based on whole-genome analysis defined 24 RSV-A lineages and 16 RSV-B lineages (13).

We previously described the genomic diversity of RSV during the 2023–2024 season using an analysis of the G and F genes (14). In the current study, we optimized a long-amplicon protocol that enables recovery of full genomes for both RSV-A and RSV-B, allowing comprehensive characterization of RSV strains circulating during the 2024–2025 season. The study aimed to assess RSV subtype prevalence and seasonal patterns, examine amino acid polymorphisms across surface and internal proteins, and evaluate clinical and demographic associations with infection severity. To our knowledge, this is the first report of comprehensive whole-genome RSV surveillance from the state of Maryland.

## MATERIALS AND METHODS

### Study specimens, nucleic acid extraction, and real-time RT-PCR

Standard-of-care RSV diagnostic tests used in the Johns Hopkins Health System (JHHS) include the Cepheid Xpert Xpress SARS-CoV-2/Flu/RSV test or the GenMark Dx ePlex RP1/RP2 respiratory panels (15, 16), both of which target the nucleocapsid (N) gene. Nucleic acid was extracted from 300 µL of each specimen and eluted to 60 µL using the Chemagic Viral DNA/RNA 360 Kit (Revvity). A research-use-only real-time RT-PCR targeting the RSV matrix (M) gene was conducted using Luna Universal Probe One-Step RT-qPCR Kit (E3006) to determine the cycle threshold (Ct) values (17).

### Primer design and two-step multiplex RT-PCR

Consensus full-length RSV-A and RSV-B genomes were obtained from GenBank to guide primer design. Primers were designed for four overlapping long-amplicons targeting both RSV-A and RSV-B genomes. First-strand cDNA synthesis was performed using 2 µL of LunaScript RT SuperMix (New England Biolab) and 8 µL of extracted RNA (thermocycling conditions: 2 min at 25°C, 10 min at 55°C, and 1 min at 95°C). Two multiplex PCR reactions (Pool 1 and Pool 2) were used to amplify the cDNA. Each 25 µL PCR reaction contained 5 µL of cDNA, 12.5 µL of Q5 Hot Start High-Fidelity 2× Master Mix, 3.5 µL of nuclease-free water, and primers at either 200 or 400 nM final concentration (achieved by adding 0.5 or 1.0 µL of each 10 µM primer, respectively). Thermocycling conditions were initial denaturation at 98°C for 30 s, 40 cycles of 98°C for 10 s, 50°C for 30 s, and 72°C for 4 min, and a final extension at 72°C for 10 min. Table S2 outlines the primer and probe sequences for real-time RT-PCR and multiplex RT-PCR.

### Genome assembly and phylogenetic tree construction

Libraries were prepared using the NEBNext ARTIC Library Prep Kit and the Oxford Nanopore Native Barcoding Kit 96 (v.14) and sequenced on an Oxford Nanopore GridION device. The resulting FASTQ files were processed using our in-house analysis pipeline (14). Closest reference genomes were identified via BLAST searches (MH447951 for RSV-A and OP975389 for RSV-B), and draft genomes were assembled using the mini_assemble module in Pomoxis. Consensus polishing was performed with Medaka, and sequencing depth was assessed using Samtools. Quality control filters were applied to retain only sequences with quality scores between 30 and 90 or higher. Low-quality or incomplete genomes were associated with higher average Ct values and were manually excluded after sequencing. RSV clade assignments were performed using Nextclade (v.3.15.3), which classifies sequences based on whole-genome phylogenetic placement. This tool assigns clade designations consistent with current genomic surveillance nomenclature, incorporating both sublineages and G-clade information. Sequences were aligned with MAFFT (v.7). Phylogenetic trees were generated using the maximum likelihood method in IQ-Tree (v.2.4.0) with 1,000 bootstrap replicates. Trees were visualized using FigTree (v.1.4.4). Reference genomes used for the phylogenetic analysis are listed in Table S3.

### Amino acid sequence analysis and glycosylation prediction

RSV-A sequences were aligned and compared to the reference genome hRSV/A/England/397/2017 (EPI_ISL_412866) due to its well-characterized full-length sequence representative of the globally dominant ON1 genotype (13). Prefusion (Protein Data Bank [PDB]: 5UDE) and post-fusion (PDB: 3RRR) F protein structures from the PDB were visualized with PyMOL (v.3.1). Amino acid variants were identified in the F protein at frequencies of ≥1% and in other viral proteins at frequencies of ≥10%. N-linked glycosylation sites (defined by the N–X–S/T motif, where X is not proline) were predicted with NetNGlyc 1.0 (threshold ≥0.5), and O-linked glycosylation sites (serine/threonine residues) were predicted with NetOGlyc 4.0 (18, 19). All substitutions are detailed in Table S4.

### Statistical analysis

Clinical and demographic data were collected for RSV-positive patients in bulk. Odds ratios (ORs) and 95% confidence intervals (CIs) were calculated using Microsoft Excel and mapped by Python to assess associations between clinical variables and outcomes (hospital admission, supplemental oxygen use, and intensive care unit [ICU]-level care), and statistical significance was defined as *P* < 0.05. Calculated odds ratios used for the forest plot are provided in Table S5.

## RESULTS

### RSV prevalence and cohort characteristics

**Fig 1:**
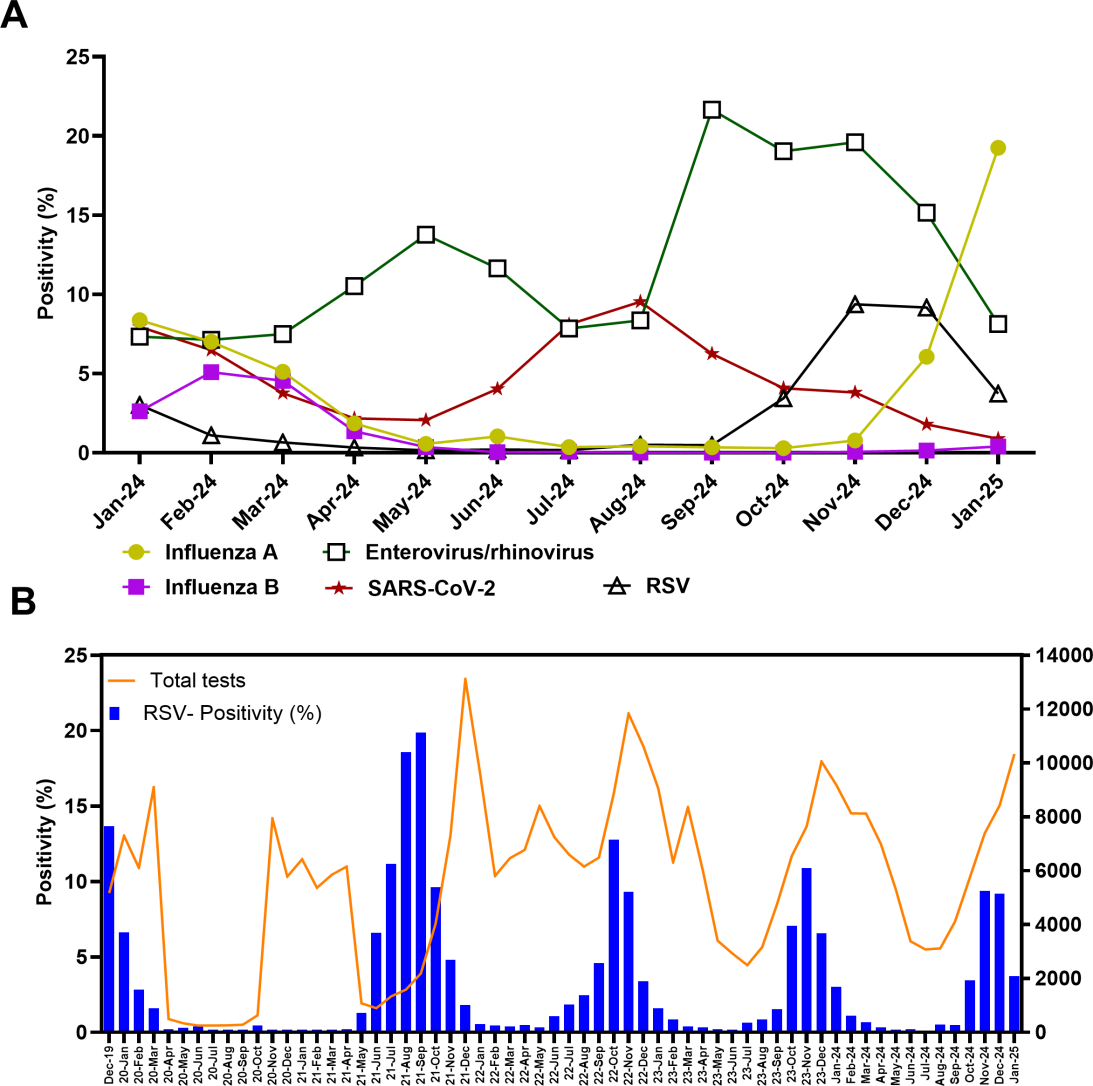
Respiratory virus circulation within JHHS. (**A**) Positivity rates of respiratory viruses between January 2024 and January 2025. (**B**) Monthly testing volume and RSV positivity rates among patients from December 2019 to January 2025.

Between June 2024 and January 2025, enterovirus/rhinovirus, influenza, RSV, and severe acute respiratory syndrome coronavirus 2 (SARS-CoV-2) exhibited the highest positivity rates in JHHS (Fig. 1A). Enterovirus/rhinovirus peaked at 21.65% in September, SARS-CoV-2 at approximately 9.53% in August, and RSV at 9.37% in November and 9.17% in December (Fig. 1B).

Between June 2024 and January 2025, 4.6% (2,096 out of 45,567) of respiratory tests performed within the JHHS were positive for RSV. We randomly sequenced 431 of these specimens, yielding 336 sequences suitable for analysis. RSV-A accounted for the majority (319 out of 336, 94.9%) of sequences (Table 1). The male-to-female ratio was 1.0:1.06, and over half of the patients (189 out of 336, 56.2%) were young children aged 1–5 years (median age: 2 years). Comorbidities were common, particularly lung disease (58 out of 336, 17.3%) and cancer (46 out of 336, 13.7%). Co-infections were identified in 6% (20 out of 336) of patients, most frequently with enterovirus/rhinovirus (13 out of 20, 65%). The majority of patients (320 out of 336, 95.2%) were seen in the emergency department; 20.2% (68 out of 336) required hospitalization; 15.8% (53 out of 336) received supplemental oxygen; and 4.5% (15 out of 336) required ICU-level care.

**TABLE 1:** Patient demographics and clinical characteristics

Characteristics	Number of patients (% cohort)		
	Cohort	RSV-A	RSV-B
Size	336	319 (94.9)	17 (5.1)
Gender			
Female	173 (51.5)	163 (51.1)	10 (58.8)
Male	163 (48.5)	156 (48.9)	7 (41.2)
Age			
0–11 months	90 (26.8)	83 (26.0)	7 (41.2)
1–5 years	189 (56.2)	181 (56.7)	8 (47.0)
6–17 years	33 (9.8)	33 (10.4)	0
18–59 years	20 (6.0)	18 (5.6)	2 (11.8)
≥60 years	4 (1.2)	4 (1.3)	0
Comorbidities			
Asthma	21 (6.3)	21 (6.6)	0
Atrial fibrillation	4 (1.2)	4 (1.3)	0
Cancer	46 (13.7)	43 (13.5)	3 (17.6)
Cerebrovascular disease	7 (2.1)	6 (1.9)	1 (5.9)
Coronary artery disease	16 (4.8)	16 (5.0)	0
Diabetes	10 (3.0)	10 (3.1)	0
Heart failure	8 (2.4)	7 (2.2)	1 (5.9)
Hypertension	24 (7.1)	24 (7.5)	0
Immunosuppression	41 (12.2)	41 (12.9)	0
Kidney disease	16 (4.8)	16 (5.0)	0
Non-asthmatic lung disease	58 (17.3)	57 (17.9)	1 (5.9)
Smoker	7 (2.1)	7 (2.2)	0
Pregnant	1 (0.3)	1 (0.3)	0
Co-infection	20 (6.0)	19 (6.0)	1 (5.9)
Emergency department visit	320 (95.2)	303 (95.0)	17 (100)
Admitted	68 (20.2)	64 (20.1)	4 (23.5)
0–11 months	19 (21.1)	16 (19.3)	3 (42.9)
1–5 years	28 (14.8)	27 (14.9)	1 (12.5)
6–17 years	10 (50.0)	10 (30.3)	0
18–59 years	8 (40.0)	8 (44.4)	0
≥60 years	3 (75.0)	3 (75.0)	0
ICU-level care	15 (4.5)	14 (4.4)	1 (5.9)
Supplemental oxygen	53 (15.8)	50 (15.7)	3 (17.6)

**Fig 2:**
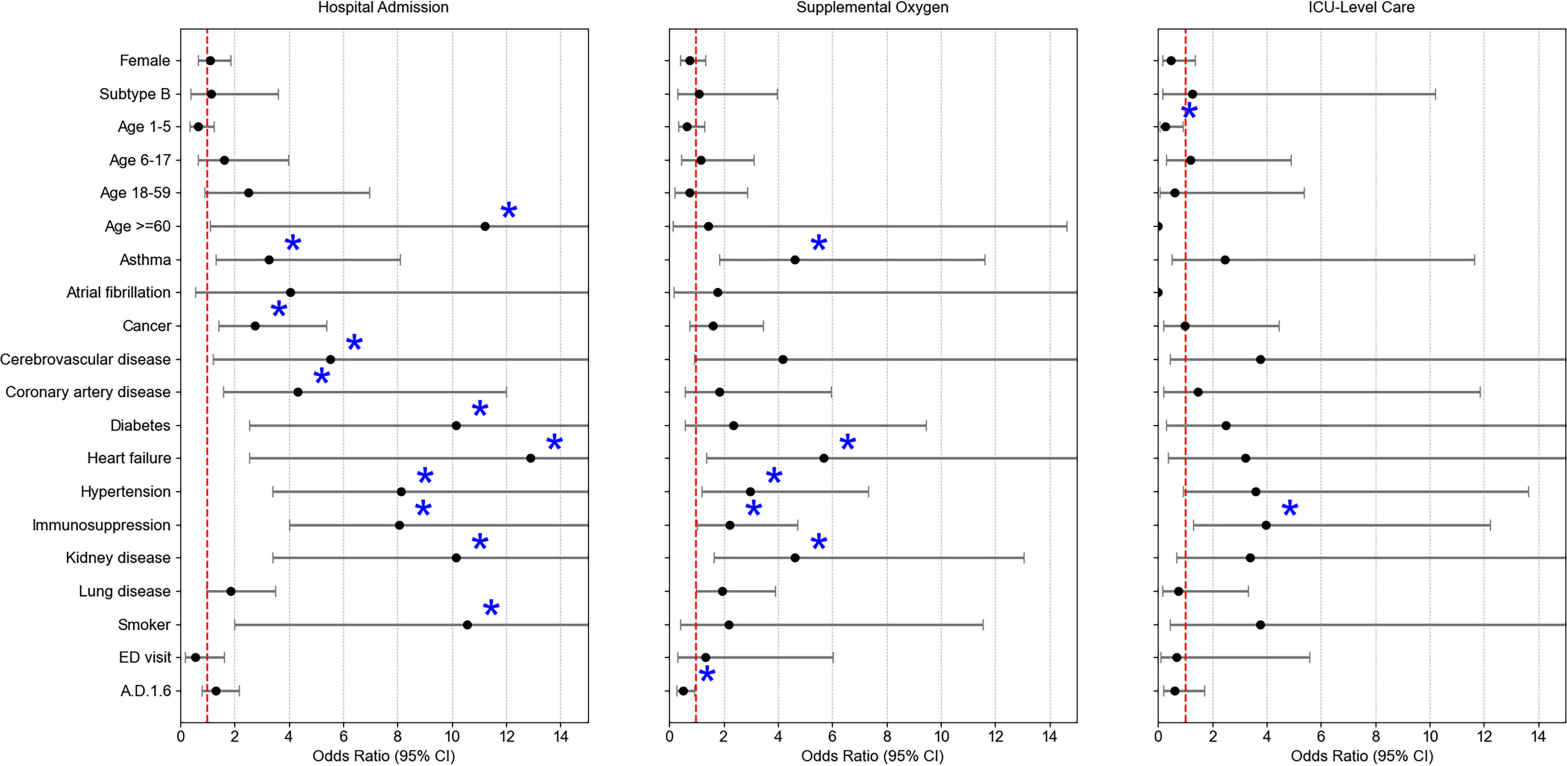
Adjusted ORs with 95% CI from the univariate logistic regression model for three clinical outcomes: hospital admission, supplemental oxygen use, and ICU-level care for RSV-positive patients. Blue asterisks indicate statistically significant associations (*P* < 0.05). Red dashed vertical lines mark the null value (OR = 1).

Associations between comorbidities and outcomes, including hospital admission, supplemental oxygen use, and ICU-level care, were evaluated (Fig. 2). Certain comorbidities were significantly associated with increased odds of admission, such as diabetes (OR: 10.13, 95% CI: 2.55-40.33; *P* = 0.001). For supplemental oxygen use, significant associations were observed with comorbidities such as heart failure (OR: 5.69, 95% CI: 1.38-23.53; *P* = 0.02). Regarding ICU-level care, only immunosuppression was significantly associated with increased odds of ICU admission (OR: 3.96, 95% CI: 1.28-12.23; *P* = 0.02).

### Full genome phylogenetic analysis

**Fig 3:**
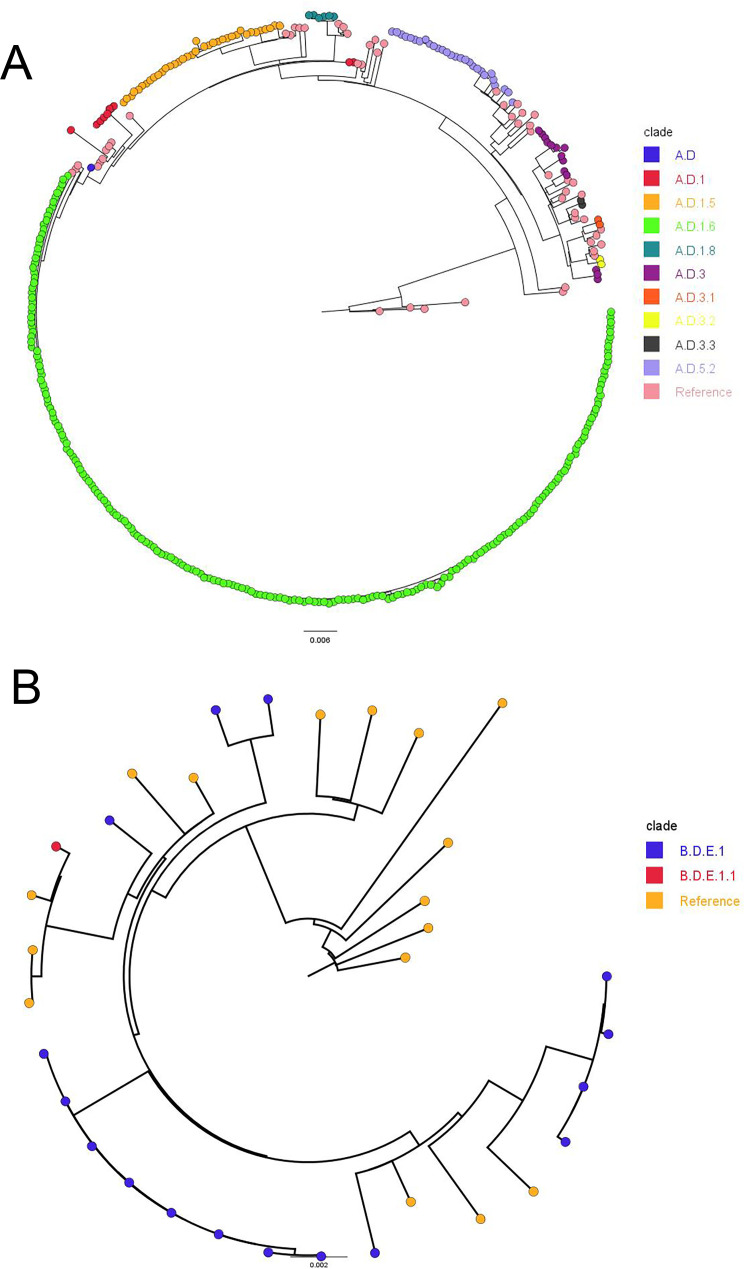
RSV clade distribution and phylogenetic trees. (**A**) Whole-genome phylogenetic tree of RSV-A, with reference genomes highlighted in light pink. (**B**) Whole-genome phylogenetic tree of RSV-B, with reference genomes highlighted in yellow-orange.

A total of 10 RSV-A clades were identified this season, with A.D.1.6 (218 out of 319, 68.34%) being predominant (Fig. 3A). RSV-A phylogeny showed tight clustering within this dominant clade. Other RSV-A clades also clustered together with their respective reference clade genomes from different geographical locations and years (Fig. 3A), except for A.D.1 and A.D.3, which formed two and three distinct branches, respectively. Only two RSV-B clades were observed (B.D.E.1 and B.D.E.1.1), with most RSV-B genomes classified as B.D.E.1 (16 out of 17, 94.12%) with four distinct phylogenetic branches (Fig. 3B). All RSV-A sequences were classified as belonging to the ON1 genotype, corresponding to G-clade GA2.3.5, and all RSV-B sequences belonged to the BA9 genotype, corresponding to G-clade GB5.0.5a. The JHHS sequences clustered with RSV genomes from Europe (Ukraine, Spain, Norway, Italy, and France) and Mexico within this season. Notably, individual-level travel history was not available, limiting our ability to determine whether these introductions were associated with international travel.

### G and F amino acid polymorphisms

**Fig 4:**
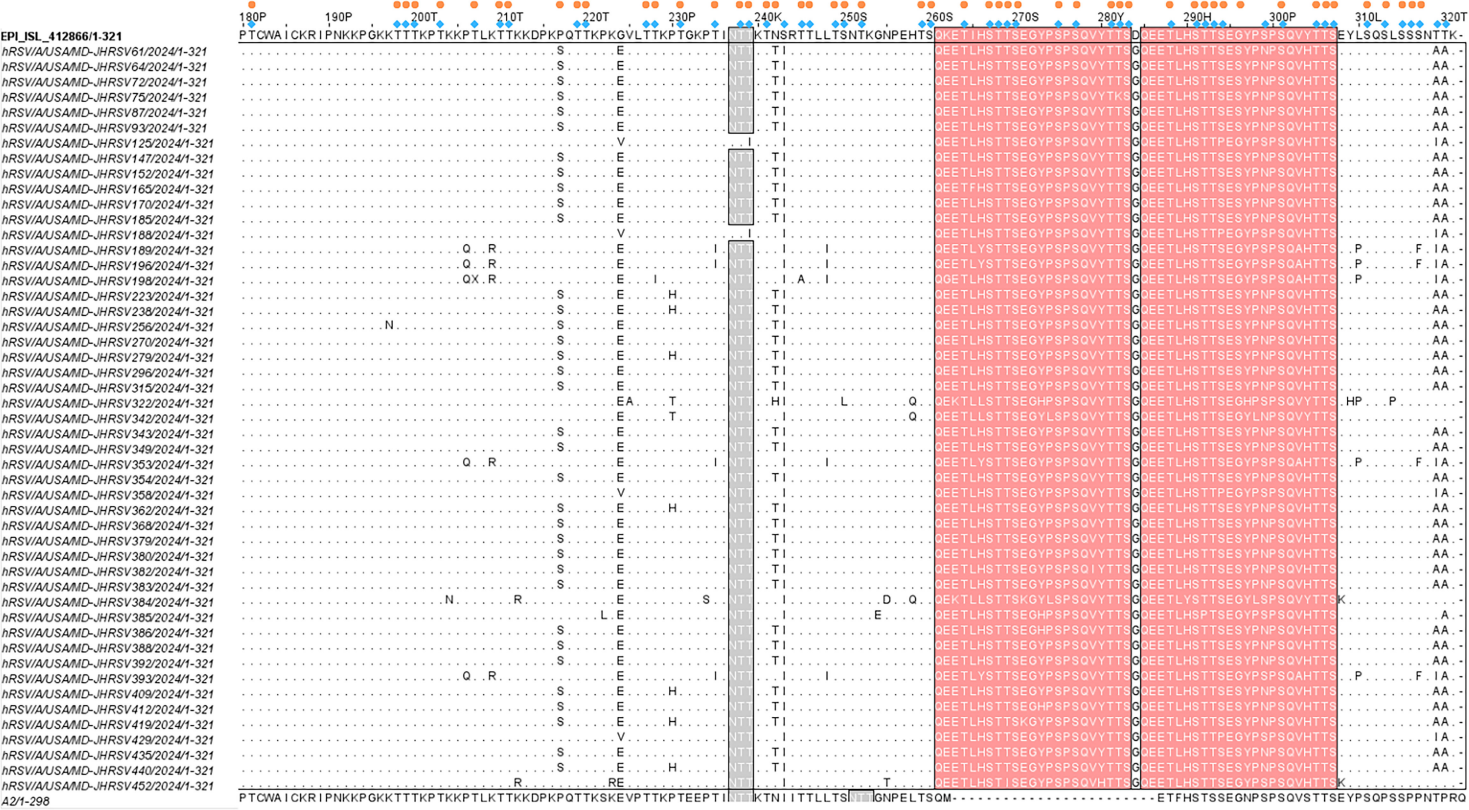
Amino acid alignment of the G protein HVR2 of RSV-A genomes. Alignment is shown relative to the reference sequence. The amino acids corresponding to G protein positions 180–320 are shown. Identical residues are indicated by dots. Outlined pink shading rectangles represent two copies of the duplicated 23 amino acids (QEETLHSTTSEGYLSPSQVYTTS) in the characteristic ON1 RSV-A genomes (20). Gray shading highlights the predicted N-glycosylation sites. Blue rhombus indicates the predicted O-glycosylation sites of the reference genome, and orange circles indicate the predicted O-glycosylation sites of our genomes. An A2 prototype genome (GenBank accession number M11486.1) was included to highlight the ON1 characteristic amino acid duplication.

We identified 28 amino acid substitutions in the G protein compared to the reference genome (Table 2), with the majority (16 out of 28, 57.1%) located in the second hypervariable region (HVR2). Two substitutions, I134K and D284G, were present in all sequences. Additionally, we observed the A57T substitution (11.04%) within the transmembrane domain, which was not detected in the previous season. The reference G protein contains three predicted N-linked glycosylation sites at positions 103, 135, and 237. In our sequences, the substitution S100N (11.1%) introduced an additional N-linked glycosylation site, while T137K (11.6%) resulted in the loss of one. O-linked glycosylation predictions in 50 randomly selected RSV-A sequences identified 50 predicted O-glycosylation sites in HVR2 (compared to 51 in the reference, including 10 in the duplicated region) (Fig. 4). The substitutions P217S (68.47%) and G296S (68.79%) added O-glycosylation sites in 16.0% (8 out of 50) and 70.0% (35 out of 50) of selected sequences, respectively; G296S lies within the duplication region. In contrast, the substitution S299N (69.52%), T319A/I, and T320A (91.43%) collectively led to the loss of three predicted O-glycosylation sites, including S299N in the duplication region.

**TABLE 2:** Amino acid substitutions identified in the RSV G and F proteins of RSV-A

G gene			F gene		
Antigenic site/structural domain	Amino acid substitutions	Frequency (%)	Antigenic site/structural domain	Amino acid substitutions	Frequency (%)
Cytoplasmic 1–37	–^*a*^	–	Ø 62–96 195–227 (nirsevimab)	–	–
Transmembrane domain 38–66	A57T	11.04	I 27–45 312–318 378–389	–	–
First hypervariable region 67–160	P71L H90Y S100N L101F G106E P120L S121I I134K T137K L142S P143S	99.69 99.37 11.08 98.75 71.34 68.89 68.89 100.00 11.64 84.76 68.79	II 254–277 (palivizumab)	K272N S276N	4.44 2.20
Conserved domain 161–200	–	–	III 46–54 301–311 345–352 367–378	S377N	2.52
Second hypervariable region 192–322	P217S G224E G224V P230H N242T S243I K262E I265L D284G S294P G296S S299N Y304H T319A T319I T320A	68.47 88.92 11.08 10.69 68.79 99.37 99.37 99.68 100.00 11.08 68.79 69.52 94.30 69.45 19.94 91.43	IV 422–471	–	–
			V 55–61 146–194 287–300	I59V	7.23
			SP 1–25	A10T T12I T13A C21W	7.23 5.99 1.89 2.52
			p27 109–136	T122A N124T V127I	8.81 7.23 11.04
			TM 530–549	–	–
			Undefined sites	S99N A103T	11.67 8.81

^*a*^ “–” indicates that no amino acid changes were identified.

**Fig 5:**
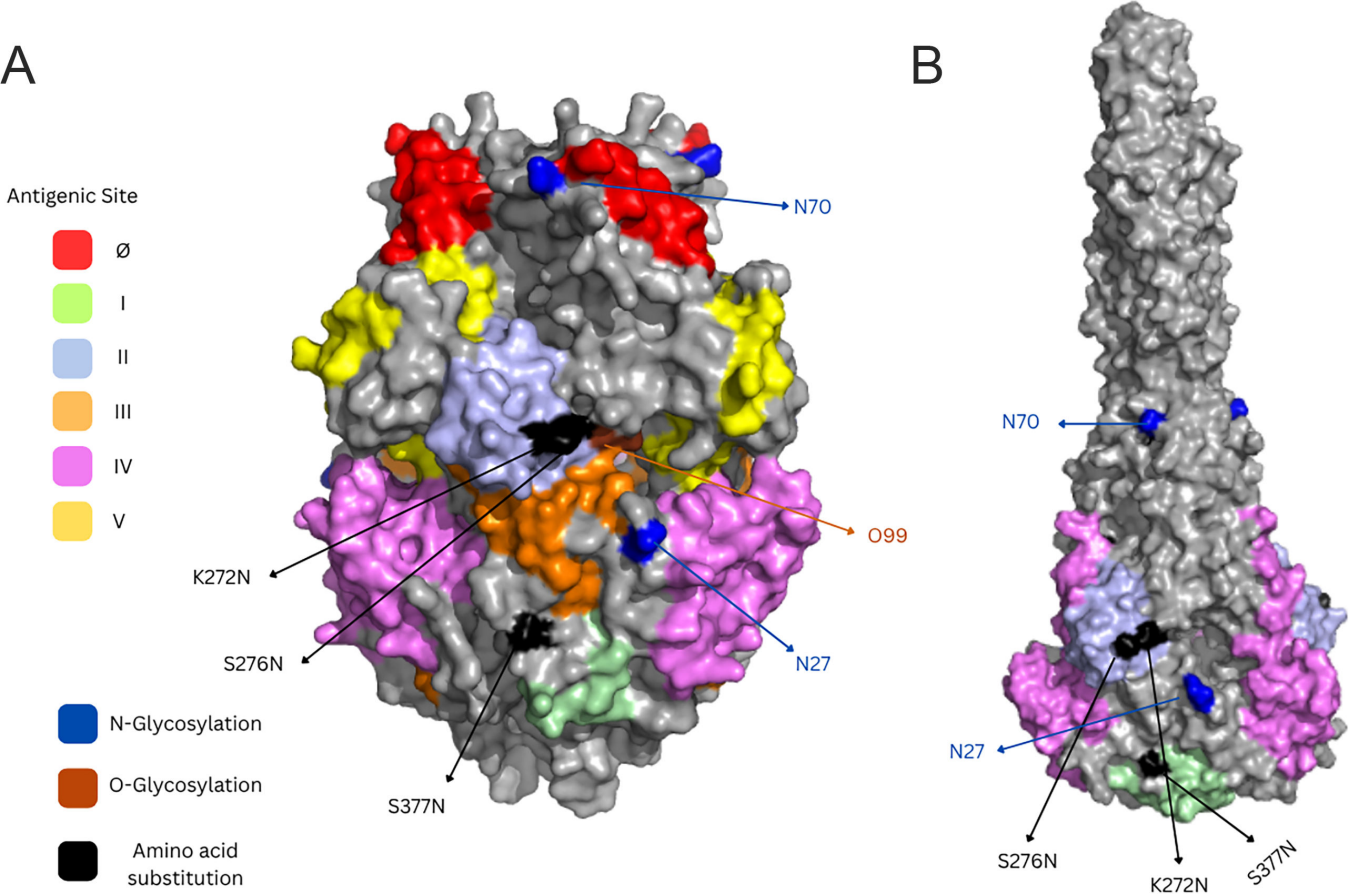
Structural mapping of amino acid substitutions on antigenic sites of the RSV F protein in (**A**) prefusion (PDB: 5UDE) and (**B**) post-fusion (PDB: 3RRR) conformations. Surface-exposed antigenic sites Ø, I, II, III, IV, and V are colored distinctly, and amino acid substitutions with a frequency of >1.0% are labeled in black. N-linked (blue) and O-linked (orange) glycosylation sites are indicated. Several amino acid residues could not be visualized due to unresolved regions in the crystal structures.

In the F protein, 13 amino acid substitutions were identified compared to the reference genome. Two substitutions, K272N (4.4%) and S276N (2.2%), were located within antigenic site II (Fig. 5). The reference F protein contains five N-linked glycosylation sites at positions 27, 70, 116, 120, and 126. The substitution T122A (8.8%) resulted in the loss of one N-linked glycosylation site, and no substitutions introduced new N-glycosylation sites. Among the same 50 randomly selected sequences, no O-linked glycosylation sites were predicted in the reference genome, but six sites were predicted in our sequences at positions 99, 105, 118, 128, 244, and 248, each appearing at different frequencies. In total, 12.2% (39 out of 319) of sequences gained at least one O-glycosylation site, with the majority (87.2%, 34 out of 39) having a predicted site at position 244. In some RSV genomes, F substitutions (R553K: 6 out of 319, 1.88%; S554N: 21 out of 319, 6.58%) were seen as mixed populations and were not captured in the consensus sequence, leading to ambiguous base calls (N).

### Amino acid polymorphisms in other proteins

**Fig 6:**
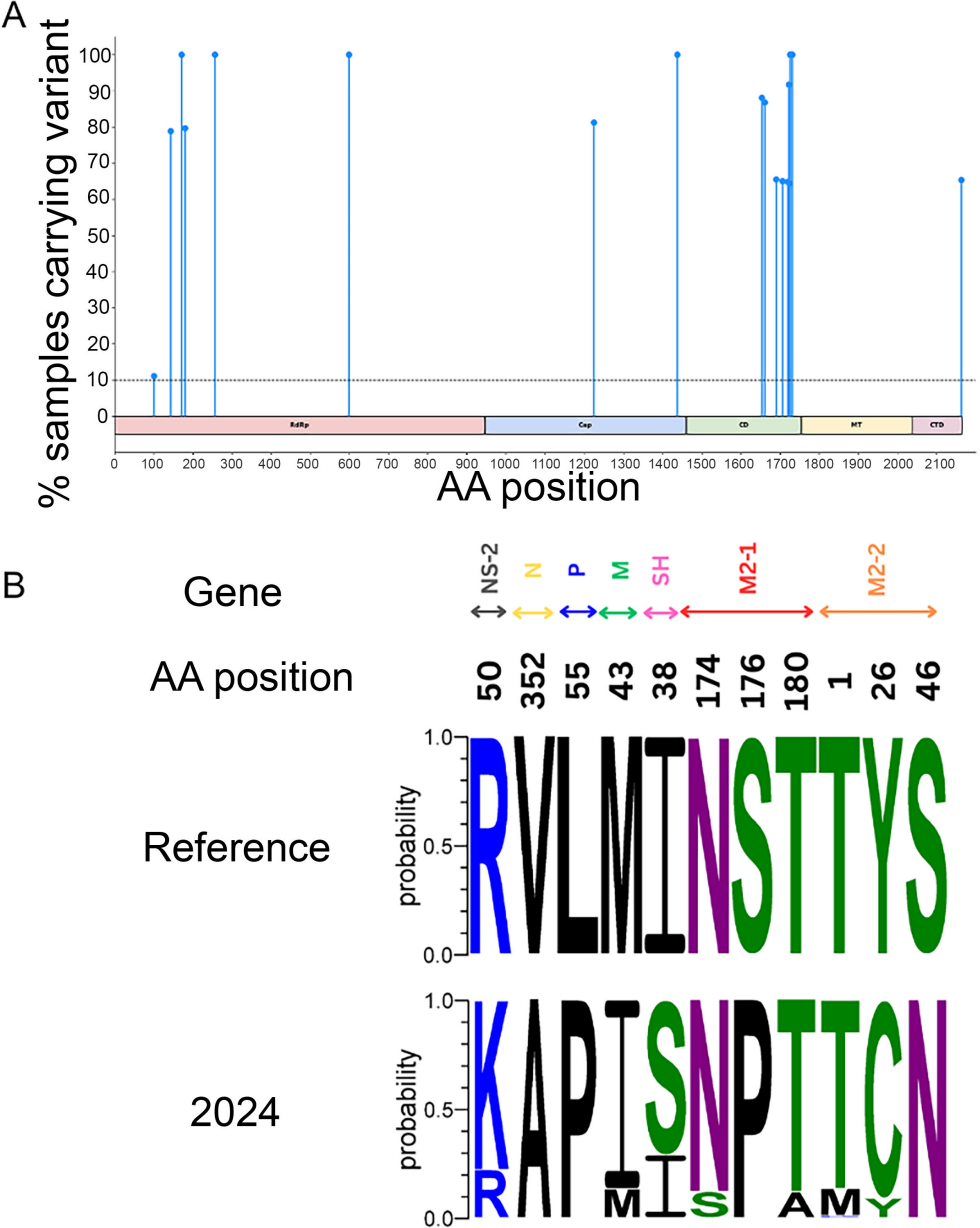
(**A**) Proportion of samples with amino acid substitutions in the RSV-A L protein compared to the reference genome. Domains shown include RNA-dependent RNA polymerase (RdRp), capping (Cap), connector domain (CD), methyltransferase (MT), and C-terminal domain (CTD). (**B**) Amino acid polymorphisms in RSV proteins—excluding F, G, and L—identified in complete RSV-A genomes from 2024, relative to the reference genome. Proteins shown include non-structural protein 2 (NS2), nucleoprotein (N), phosphoprotein (P), matrix (M), small hydrophobic protein (SH), and transcription antitermination proteins (M2-1 and M2-2). Non-structural protein 1 is not shown, as no substitutions were detected.

In the polymerase (L) protein, we identified 18 amino acid substitutions spanning all domains compared to the reference genome (Fig. 6A). Half of these (9 out of 18, 50%) clustered within the connector domain (CD) domain, primarily between amino acid positions 1,653 and 1,731, and were present at high frequencies. One-third of the substitutions (6 out of 18, 33.3%) were located in the RNA-dependent RNA polymerase (RdRp) domain. The Cap domain and CTD had two and one substitutions, respectively, while no substitutions were observed in the MT domain. Several L protein substitutions, P171L, R256K, Y598H, L1438Q, E1725G, and G1731D, were fixed in all sequences. Substitutions were also identified in the M, M2-1, M2-2, NS2, P, SH, and N proteins, whereas none were detected in NS1 compared to the reference genome (Fig. 6B). Notably, several substitutions became fixed in all our sequences, including V352A in the N protein, L55P in the P protein, S176P in M2-1, and S46N in M2-2.

## DISCUSSION

Public health measures implemented during the COVID-19 pandemic significantly reduced the prevalence and genetic diversity of respiratory viruses during the 2020–2021 seasons (21). As these measures were relaxed, RSV resurged with an atypical early fall peak globally (20, 22, 23), likely driven by reduced population immunity (24, 25). Although global antibody recovery was observed following the resurgence (26), some studies reported no significant differences in population-level RSV immunity between the prepandemic and post-pandemic periods (27, 28). During the 2024–2025 season, RSV activity increased in late 2024 and peaked in January 2025, reflecting a gradual return to prepandemic seasonal patterns (29–31). This season was marked by RSV-A predominance, in contrast to the previous season’s RSV-B dominance, consistent with global subtype shifts influenced by herd immunity dynamics (32, 33). However, RSV seasonality continues to vary across national and regional levels (34), and other studies in the U.S.A. observed RSV-B predominance during the same season (35).

In our cohort, children aged 1–5 years accounted for the majority of RSV cases, despite national and JHHS data indicating higher positivity rates among infants and older adults (36, 37). Notably, this age group currently lacks access to prophylactic interventions and may experience reduced protection from maternally derived immunity (38). The predominant RSV-A clade, A.D.1.6, was responsible for most hospitalizations, although no significant associations with clinical severity were observed. While our univariate logistic regression analyses suggested potential associations between comorbidities and clinical outcomes, the limited sample size constrains the strength of these conclusions.

Our phylogenetic analysis revealed regional expansion of clade A.D.1.6, a sublineage of the globally dominant ON1 genotype of RSV-A, which is characterized by a 72-nucleotide duplication in the G gene. This expansion is likely driven by immune evasion and altered transmissibility, as observed in SARS-CoV-2 evolution (39, 40). All sequences clustered within this clade, suggesting limited intra-clade genetic diversity. The continued prevalence of the parent clade A.D.1 during the previous season may have facilitated the emergence of A.D.1.6 through the accumulation of fixed substitutions. These genetic changes may have conferred a selective advantage, allowing A.D.1.6 to outcompete other RSV-A clades during the current season (41). Interestingly, no reports of A.D.1.6 dominance have emerged globally this season. Smaller clusters representing clades A.D.1, A.D.3, and B.D.E.1 appear to have arisen through the gain or loss of key substitutions, resulting in their assignment to existing clades by Nextclade while forming distinct clusters in our phylogenetic analysis. For RSV-B, we observed limited genetic diversity, with most sequences falling within the B.D.E.1 clade, which is derived from the globally circulating BA9 genotype carrying the hallmark 60-nucleotide G gene duplication. The reduced representation of RSV-B in our data set—compared to previous seasons when BA9-derived lineages were more prevalent within JHHS—may reflect a recent bottleneck event (42), although undersampling or geographical restriction cannot be ruled out. We also detected a few B.D.E.1 cases, a clade dominant in the prior season, indicating potential epidemiological relevance. This observation aligns with a recent B.D.E.1 surveillance report from Beijing (43).

We found that the set of HVR1 and HVR2 G protein substitutions prevalent in previous seasons persisted at high frequency (>88%) during the current season (14). Notably, substitutions such as P71L, H90Y, I134K, and S243I have been associated with severe acute respiratory infections in pediatric patients (44). Although limited information is available regarding the impact of substitutions in the transmembrane domain, the emergence of A57T and several other changes in this region have been shown to reduce the affinity of neutralizing antibodies (45). The observed gains and losses of glycosylation sites likely reflect viral adaptation under immune pressure (46, 47). For instance, newly introduced glycosylation sites may help mask immunodominant epitopes (48), while the loss of specific glycans could enhance antigen presentation and T-cell activation (49). These ongoing changes in substitutions and glycosylation patterns in the G protein highlight the need for continued genomic surveillance.

In the F protein, we tracked amino acid substitutions occurring at a frequency of ≥1% due to their relevance for vaccines and monoclonal antibody therapies (50). All four substitutions detected during the previous season persisted into the current season (14). A new substitution, K272N, along with S276N, lies within antigenic site II, the target of Palivizumab. K272N has been shown to impair palivizumab neutralization (51), while S276N and S377N are located within immunodominant regions of the F protein (52).

We observed the loss of a conserved N-linked glycosylation site in some F sequences due to the T122A substitution, which may impact immune recognition (49). Although the reference F protein lacks known O-linked glycans, some of our genomes contain amino acid changes that are predicted to introduce potential O-glycosylation sites, which could possibly contribute to shielding of neutralizing epitopes (53). Importantly, none of the substitutions in our sequences affected the specific residues engineered in the new RSV F vaccines (S155C, S190F, V207L, and S290C), suggesting that vaccine-targeted epitopes remain conserved (54). However, a recent study reported limited evidence of antigenic drift in the F protein during the first season following vaccine introduction (55).

The high-frequency clustering of L protein substitutions in the RdRp and CD domains may impact viral replication and sensitivity to antivirals (56). These changes could potentially influence the binding of nucleoside analog inhibitors such as ribavirin (57), although structural insights into the RdRp and CD regions in the context of these nucleotide analogs remain limited (58). Notably, none of the previously reported resistance-associated substitutions in the CD domain (Y1631H/C, L1502Q, and H1632Q), which have been linked to reduced antiviral potency, were found in our sequences (59). During initial sequencing, one amplicon covering L residues 889–2,166 showed low coverage, which we corrected by adjusting primer concentrations. Finally, all substitutions identified in the other RSV proteins were previously reported in Global Initiative on Sharing All Influenza Data and have been observed globally (44). The fixation of some of these changes in our genomes highlights the need for further functional investigation.

This study has several limitations, including the small number of RSV-B samples, which limited subtype comparisons, the lack of assessment for RSV-A/B co-infections, and potential sample collection bias due to clinical testing being performed primarily in certain groups or symptomatic patients. Additionally, all glycosylation-related findings are based on *in silico* predictions and were not experimentally validated. Individual-level data on prior RSV exposure or administration of antivirals and monoclonal antibodies were not available, limiting our ability to directly assess the impact of immune pressure on viral evolution. Nonetheless, our findings highlight the realignment of RSV seasonality in Maryland and underscore the value of whole-genome surveillance in detecting emerging clades and molecular variation.

## Data Availability

RSV genomes are available in the Global Initiative on Sharing All Influenza Data database (Table S1).

## SUPPLEMENTAL MATERIAL

Table S1 spectrum.02065-25-s0001.docx RSV genomes GISAID accession numbers.

Table S2 spectrum.02065-25-s0002.docx Primers and probes used for RSV amplification and detection.

Table S3 spectrum.02065-25-s0003.docx Reference genomes used for the phylogenetic analysis.

Table S4 spectrum.02065-25-s0004.docx Amino acid substitutions identified in RSV sequences.

Table S5 spectrum.02065-25-s0005.docx Calculated odds ratios used for the forest plot analyses.

ASM does not own the copyrights to Supplemental Material that may be linked to, or accessed through, an article. The authors have granted ASM a non-exclusive, world-wide license to publish the Supplemental Material files. Please contact the corresponding author directly for reuse.
