# Supplementary material for: Whole-genome sequence characterization of respiratory syncytial virus in the Johns Hopkins Health System during the 2024–2025 respiratory season
Source: Microbiol Spectr. 2025 Oct 7;13(11):e02065-25. doi: 10.1128/spectrum.02065-25 (PMC12584621; doi:10.1128/spectrum.02065-25)
Supplement: Table S1 — RSV genomes GISAID accession numbers. [file spectrum.02065-25-s0001.docx]

Supplementary Table S1. GISAID sequence accession numbers of RSV-A and RSV-B strains in this study

| **RSV strains** | **Accession ID** |
| --- | --- |
| hRSV/A/USA/MD-JHSOM460/2024 | EPI_ISL_19819188 |
| hRSV/B/USA/MD-JHSOM459/2024 | EPI_ISL_19819187 |
| hRSV/A/USA/MD-JHSOM458/2024 | EPI_ISL_19819186 |
| hRSV/B/USA/MD-JHSOM457/2024 | EPI_ISL_19819185 |
| hRSV/A/USA/MD-JHSOM456/2024 | EPI_ISL_19819184 |
| hRSV/A/USA/MD-JHSOM455/2024 | EPI_ISL_19819183 |
| hRSV/A/USA/MD-JHSOM454/2024 | EPI_ISL_19819182 |
| hRSV/A/USA/MD-JHSOM453/2024 | EPI_ISL_19819181 |
| hRSV/A/USA/MD-JHSOM452/2024 | EPI_ISL_19819180 |
| hRSV/B/USA/MD-JHSOM451/2024 | EPI_ISL_19819179 |
| hRSV/A/USA/MD-JHSOM450/2024 | EPI_ISL_19819178 |
| hRSV/A/USA/MD-JHSOM448/2024 | EPI_ISL_19819177 |
| hRSV/A/USA/MD-JHSOM447/2024 | EPI_ISL_19819176 |
| hRSV/A/USA/MD-JHSOM446/2024 | EPI_ISL_19819175 |
| hRSV/A/USA/MD-JHSOM445/2024 | EPI_ISL_19819174 |
| hRSV/A/USA/MD-JHSOM444/2024 | EPI_ISL_19819173 |
| hRSV/A/USA/MD-JHSOM443/2024 | EPI_ISL_19819172 |
| hRSV/A/USA/MD-JHSOM442/2024 | EPI_ISL_19819171 |
| hRSV/A/USA/MD-JHSOM440/2024 | EPI_ISL_19819170 |
| hRSV/A/USA/MD-JHSOM439/2024 | EPI_ISL_19819169 |
| hRSV/A/USA/MD-JHSOM438/2024 | EPI_ISL_19819168 |
| hRSV/B/USA/MD-JHSOM437/2024 | EPI_ISL_19819167 |
| hRSV/A/USA/MD-JHSOM435/2024 | EPI_ISL_19819166 |
| hRSV/A/USA/MD-JHSOM434/2024 | EPI_ISL_19819165 |
| hRSV/A/USA/MD-JHSOM433/2024 | EPI_ISL_19819164 |
| hRSV/A/USA/MD-JHSOM432/2024 | EPI_ISL_19819163 |
| hRSV/A/USA/MD-JHSOM431/2024 | EPI_ISL_19819162 |
| hRSV/A/USA/MD-JHSOM430/2024 | EPI_ISL_19819161 |
| hRSV/A/USA/MD-JHSOM429/2024 | EPI_ISL_19819160 |
| hRSV/A/USA/MD-JHSOM428/2024 | EPI_ISL_19819159 |
| hRSV/A/USA/MD-JHSOM427/2024 | EPI_ISL_19819158 |
| hRSV/A/USA/MD-JHSOM426/2024 | EPI_ISL_19819157 |
| hRSV/A/USA/MD-JHSOM425/2024 | EPI_ISL_19819156 |
| hRSV/A/USA/MD-JHSOM424/2024 | EPI_ISL_19819155 |
| hRSV/A/USA/MD-JHSOM423/2024 | EPI_ISL_19819154 |
| hRSV/A/USA/MD-JHSOM422/2024 | EPI_ISL_19819153 |
| hRSV/A/USA/MD-JHSOM421/2024 | EPI_ISL_19819152 |
| hRSV/A/USA/MD-JHSOM419/2024 | EPI_ISL_19819151 |
| hRSV/A/USA/MD-JHSOM418/2024 | EPI_ISL_19819150 |
| hRSV/A/USA/MD-JHSOM416/2024 | EPI_ISL_19819149 |
| hRSV/A/USA/MD-JHSOM415/2024 | EPI_ISL_19819148 |
| hRSV/A/USA/MD-JHSOM413/2024 | EPI_ISL_19819147 |
| hRSV/A/USA/MD-JHSOM412/2024 | EPI_ISL_19819146 |
| hRSV/A/USA/MD-JHSOM411/2024 | EPI_ISL_19819145 |
| hRSV/A/USA/MD-JHSOM410/2024 | EPI_ISL_19819144 |
| hRSV/A/USA/MD-JHSOM409/2024 | EPI_ISL_19819143 |
| hRSV/A/USA/MD-JHSOM408/2024 | EPI_ISL_19819142 |
| hRSV/A/USA/MD-JHSOM407/2024 | EPI_ISL_19819141 |
| hRSV/A/USA/MD-JHSOM406/2024 | EPI_ISL_19819140 |
| hRSV/A/USA/MD-JHSOM405/2024 | EPI_ISL_19819139 |
| hRSV/A/USA/MD-JHSOM404/2024 | EPI_ISL_19819138 |
| hRSV/A/USA/MD-JHSOM403/2024 | EPI_ISL_19819137 |
| hRSV/A/USA/MD-JHSOM402/2024 | EPI_ISL_19819136 |
| hRSV/A/USA/MD-JHSOM401/2024 | EPI_ISL_19819135 |
| hRSV/A/USA/MD-JHSOM400/2024 | EPI_ISL_19819134 |
| hRSV/A/USA/MD-JHSOM399/2024 | EPI_ISL_19819133 |
| hRSV/A/USA/MD-JHSOM397/2024 | EPI_ISL_19819132 |
| hRSV/A/USA/MD-JHSOM396/2024 | EPI_ISL_19819131 |
| hRSV/A/USA/MD-JHSOM395/2024 | EPI_ISL_19819130 |
| hRSV/A/USA/MD-JHSOM394/2024 | EPI_ISL_19819129 |
| hRSV/A/USA/MD-JHSOM393/2024 | EPI_ISL_19819128 |
| hRSV/A/USA/MD-JHSOM392/2024 | EPI_ISL_19819127 |
| hRSV/A/USA/MD-JHSOM391/2024 | EPI_ISL_19819126 |
| hRSV/A/USA/MD-JHSOM390/2024 | EPI_ISL_19819125 |
| hRSV/A/USA/MD-JHSOM389/2024 | EPI_ISL_19819124 |
| hRSV/A/USA/MD-JHSOM388/2024 | EPI_ISL_19819123 |
| hRSV/A/USA/MD-JHSOM387/2024 | EPI_ISL_19819122 |
| hRSV/A/USA/MD-JHSOM386/2024 | EPI_ISL_19819121 |
| hRSV/A/USA/MD-JHSOM385/2024 | EPI_ISL_19819120 |
| hRSV/A/USA/MD-JHSOM384/2024 | EPI_ISL_19819119 |
| hRSV/A/USA/MD-JHSOM383/2024 | EPI_ISL_19819118 |
| hRSV/A/USA/MD-JHSOM382/2024 | EPI_ISL_19819117 |
| hRSV/A/USA/MD-JHSOM381/2024 | EPI_ISL_19819116 |
| hRSV/A/USA/MD-JHSOM380/2024 | EPI_ISL_19819115 |
| hRSV/A/USA/MD-JHSOM379/2024 | EPI_ISL_19819114 |
| hRSV/A/USA/MD-JHSOM378/2024 | EPI_ISL_19819113 |
| hRSV/A/USA/MD-JHSOM377/2024 | EPI_ISL_19819112 |
| hRSV/A/USA/MD-JHSOM375/2024 | EPI_ISL_19819111 |
| hRSV/A/USA/MD-JHSOM374/2024 | EPI_ISL_19819110 |
| hRSV/A/USA/MD-JHSOM371/2024 | EPI_ISL_19819109 |
| hRSV/A/USA/MD-JHSOM370/2024 | EPI_ISL_19819108 |
| hRSV/A/USA/MD-JHSOM368/2024 | EPI_ISL_19819107 |
| hRSV/A/USA/MD-JHSOM367/2024 | EPI_ISL_19819106 |
| hRSV/A/USA/MD-JHSOM365/2024 | EPI_ISL_19819105 |
| hRSV/A/USA/MD-JHSOM363/2024 | EPI_ISL_19819104 |
| hRSV/A/USA/MD-JHSOM362/2024 | EPI_ISL_19819103 |
| hRSV/A/USA/MD-JHSOM361/2024 | EPI_ISL_19819102 |
| hRSV/A/USA/MD-JHSOM360/2024 | EPI_ISL_19819101 |
| hRSV/A/USA/MD-JHSOM359/2024 | EPI_ISL_19819100 |
| hRSV/A/USA/MD-JHSOM358/2024 | EPI_ISL_19819099 |
| hRSV/A/USA/MD-JHSOM357/2024 | EPI_ISL_19819098 |
| hRSV/A/USA/MD-JHSOM356/2024 | EPI_ISL_19819097 |
| hRSV/A/USA/MD-JHSOM355/2024 | EPI_ISL_19819096 |
| hRSV/A/USA/MD-JHSOM354/2024 | EPI_ISL_19819095 |
| hRSV/A/USA/MD-JHSOM353/2024 | EPI_ISL_19819094 |
| hRSV/A/USA/MD-JHSOM352/2024 | EPI_ISL_19819093 |
| hRSV/A/USA/MD-JHSOM351/2024 | EPI_ISL_19819092 |
| hRSV/A/USA/MD-JHSOM350/2024 | EPI_ISL_19819091 |
| hRSV/A/USA/MD-JHSOM349/2024 | EPI_ISL_19819090 |
| hRSV/A/USA/MD-JHSOM347/2024 | EPI_ISL_19819089 |
| hRSV/A/USA/MD-JHSOM345/2024 | EPI_ISL_19819088 |
| hRSV/B/USA/MD-JHSOM344/2024 | EPI_ISL_19819087 |
| hRSV/A/USA/MD-JHSOM343/2024 | EPI_ISL_19819086 |
| hRSV/A/USA/MD-JHSOM342/2024 | EPI_ISL_19819085 |
| hRSV/A/USA/MD-JHSOM341/2024 | EPI_ISL_19819084 |
| hRSV/A/USA/MD-JHSOM340/2024 | EPI_ISL_19819083 |
| hRSV/A/USA/MD-JHSOM339/2024 | EPI_ISL_19819082 |
| hRSV/A/USA/MD-JHSOM338/2024 | EPI_ISL_19819081 |
| hRSV/B/USA/MD-JHSOM337/2024 | EPI_ISL_19819080 |
| hRSV/A/USA/MD-JHSOM336/2024 | EPI_ISL_19819079 |
| hRSV/A/USA/MD-JHSOM335/2024 | EPI_ISL_19819078 |
| hRSV/A/USA/MD-JHSOM333/2024 | EPI_ISL_19819077 |
| hRSV/A/USA/MD-JHSOM332/2024 | EPI_ISL_19819076 |
| hRSV/A/USA/MD-JHSOM331/2024 | EPI_ISL_19819075 |
| hRSV/A/USA/MD-JHSOM329/2024 | EPI_ISL_19819074 |
| hRSV/A/USA/MD-JHSOM328/2024 | EPI_ISL_19819073 |
| hRSV/A/USA/MD-JHSOM327/2024 | EPI_ISL_19819072 |
| hRSV/B/USA/MD-JHSOM326/2024 | EPI_ISL_19819071 |
| hRSV/A/USA/MD-JHSOM325/2024 | EPI_ISL_19819070 |
| hRSV/A/USA/MD-JHSOM324/2024 | EPI_ISL_19819069 |
| hRSV/A/USA/MD-JHSOM323/2024 | EPI_ISL_19819068 |
| hRSV/A/USA/MD-JHSOM322/2024 | EPI_ISL_19819067 |
| hRSV/A/USA/MD-JHSOM321/2024 | EPI_ISL_19819066 |
| hRSV/A/USA/MD-JHSOM319/2024 | EPI_ISL_19819065 |
| hRSV/A/USA/MD-JHSOM318/2024 | EPI_ISL_19819064 |
| hRSV/A/USA/MD-JHSOM317/2024 | EPI_ISL_19819063 |
| hRSV/A/USA/MD-JHSOM315/2024 | EPI_ISL_19819062 |
| hRSV/A/USA/MD-JHSOM314/2024 | EPI_ISL_19819061 |
| hRSV/A/USA/MD-JHSOM313/2024 | EPI_ISL_19819060 |
| hRSV/A/USA/MD-JHSOM311/2024 | EPI_ISL_19819059 |
| hRSV/A/USA/MD-JHSOM310/2024 | EPI_ISL_19819058 |
| hRSV/A/USA/MD-JHSOM308/2024 | EPI_ISL_19819057 |
| hRSV/A/USA/MD-JHSOM307/2024 | EPI_ISL_19819056 |
| hRSV/A/USA/MD-JHSOM306/2024 | EPI_ISL_19819055 |
| hRSV/A/USA/MD-JHSOM305/2024 | EPI_ISL_19819054 |
| hRSV/A/USA/MD-JHSOM304/2024 | EPI_ISL_19819053 |
| hRSV/A/USA/MD-JHSOM303/2024 | EPI_ISL_19819052 |
| hRSV/A/USA/MD-JHSOM302/2024 | EPI_ISL_19819051 |
| hRSV/A/USA/MD-JHSOM301/2024 | EPI_ISL_19819050 |
| hRSV/A/USA/MD-JHSOM300/2024 | EPI_ISL_19819049 |
| hRSV/A/USA/MD-JHSOM299/2024 | EPI_ISL_19819048 |
| hRSV/A/USA/MD-JHSOM297/2024 | EPI_ISL_19819047 |
| hRSV/A/USA/MD-JHSOM296/2024 | EPI_ISL_19819046 |
| hRSV/A/USA/MD-JHSOM295/2024 | EPI_ISL_19819045 |
| hRSV/A/USA/MD-JHSOM293/2024 | EPI_ISL_19819044 |
| hRSV/A/USA/MD-JHSOM292/2024 | EPI_ISL_19819043 |
| hRSV/A/USA/MD-JHSOM291/2024 | EPI_ISL_19819042 |
| hRSV/A/USA/MD-JHSOM290/2024 | EPI_ISL_19819041 |
| hRSV/A/USA/MD-JHSOM288/2024 | EPI_ISL_19819040 |
| hRSV/A/USA/MD-JHSOM287/2024 | EPI_ISL_19819039 |
| hRSV/A/USA/MD-JHSOM286/2024 | EPI_ISL_19819038 |
| hRSV/A/USA/MD-JHSOM283/2024 | EPI_ISL_19819037 |
| hRSV/A/USA/MD-JHSOM281/2024 | EPI_ISL_19819036 |
| hRSV/A/USA/MD-JHSOM280/2024 | EPI_ISL_19819035 |
| hRSV/A/USA/MD-JHSOM279/2024 | EPI_ISL_19819034 |
| hRSV/A/USA/MD-JHSOM278/2024 | EPI_ISL_19819033 |
| hRSV/A/USA/MD-JHSOM277/2024 | EPI_ISL_19819032 |
| hRSV/A/USA/MD-JHSOM275/2024 | EPI_ISL_19819031 |
| hRSV/A/USA/MD-JHSOM273/2024 | EPI_ISL_19819030 |
| hRSV/A/USA/MD-JHSOM272/2024 | EPI_ISL_19819029 |
| hRSV/A/USA/MD-JHSOM271/2024 | EPI_ISL_19819028 |
| hRSV/A/USA/MD-JHSOM270/2024 | EPI_ISL_19819027 |
| hRSV/A/USA/MD-JHSOM269/2024 | EPI_ISL_19819026 |
| hRSV/A/USA/MD-JHSOM267/2024 | EPI_ISL_19819025 |
| hRSV/B/USA/MD-JHSOM264/2024 | EPI_ISL_19819024 |
| hRSV/A/USA/MD-JHSOM262/2024 | EPI_ISL_19819023 |
| hRSV/A/USA/MD-JHSOM259/2024 | EPI_ISL_19819022 |
| hRSV/A/USA/MD-JHSOM258/2024 | EPI_ISL_19819021 |
| hRSV/A/USA/MD-JHSOM257/2024 | EPI_ISL_19819020 |
| hRSV/A/USA/MD-JHSOM256/2024 | EPI_ISL_19819019 |
| hRSV/A/USA/MD-JHSOM254/2024 | EPI_ISL_19819018 |
| hRSV/A/USA/MD-JHSOM253/2024 | EPI_ISL_19819017 |
| hRSV/A/USA/MD-JHSOM251/2024 | EPI_ISL_19819016 |
| hRSV/A/USA/MD-JHSOM250/2024 | EPI_ISL_19819015 |
| hRSV/A/USA/MD-JHSOM248/2024 | EPI_ISL_19819014 |
| hRSV/A/USA/MD-JHSOM246/2024 | EPI_ISL_19819013 |
| hRSV/A/USA/MD-JHSOM245/2024 | EPI_ISL_19819012 |
| hRSV/B/USA/MD-JHSOM243/2024 | EPI_ISL_19819011 |
| hRSV/A/USA/MD-JHSOM242/2024 | EPI_ISL_19819010 |
| hRSV/A/USA/MD-JHSOM241/2024 | EPI_ISL_19819009 |
| hRSV/A/USA/MD-JHSOM240/2024 | EPI_ISL_19819008 |
| hRSV/A/USA/MD-JHSOM239/2024 | EPI_ISL_19819007 |
| hRSV/A/USA/MD-JHSOM238/2024 | EPI_ISL_19819006 |
| hRSV/A/USA/MD-JHSOM237/2024 | EPI_ISL_19819005 |
| hRSV/A/USA/MD-JHSOM235/2024 | EPI_ISL_19819004 |
| hRSV/A/USA/MD-JHSOM232/2024 | EPI_ISL_19819003 |
| hRSV/A/USA/MD-JHSOM231/2024 | EPI_ISL_19819002 |
| hRSV/A/USA/MD-JHSOM230/2024 | EPI_ISL_19819001 |
| hRSV/A/USA/MD-JHSOM229/2024 | EPI_ISL_19819000 |
| hRSV/A/USA/MD-JHSOM227/2024 | EPI_ISL_19818999 |
| hRSV/A/USA/MD-JHSOM226/2024 | EPI_ISL_19818998 |
| hRSV/A/USA/MD-JHSOM225/2024 | EPI_ISL_19818997 |
| hRSV/A/USA/MD-JHSOM223/2024 | EPI_ISL_19818996 |
| hRSV/A/USA/MD-JHSOM222/2024 | EPI_ISL_19818995 |
| hRSV/A/USA/MD-JHSOM220/2024 | EPI_ISL_19818994 |
| hRSV/A/USA/MD-JHSOM218/2024 | EPI_ISL_19818993 |
| hRSV/A/USA/MD-JHSOM214/2024 | EPI_ISL_19818992 |
| hRSV/A/USA/MD-JHSOM212/2024 | EPI_ISL_19818991 |
| hRSV/A/USA/MD-JHSOM210/2024 | EPI_ISL_19818990 |
| hRSV/A/USA/MD-JHSOM209/2024 | EPI_ISL_19818989 |
| hRSV/A/USA/MD-JHSOM207/2024 | EPI_ISL_19818988 |
| hRSV/A/USA/MD-JHSOM206/2024 | EPI_ISL_19818987 |
| hRSV/A/USA/MD-JHSOM205/2024 | EPI_ISL_19818986 |
| hRSV/A/USA/MD-JHSOM204/2024 | EPI_ISL_19818985 |
| hRSV/A/USA/MD-JHSOM203/2024 | EPI_ISL_19818984 |
| hRSV/B/USA/MD-JHSOM202/2024 | EPI_ISL_19818983 |
| hRSV/A/USA/MD-JHSOM201/2024 | EPI_ISL_19818982 |
| hRSV/A/USA/MD-JHSOM200/2024 | EPI_ISL_19818981 |
| hRSV/A/USA/MD-JHSOM199/2024 | EPI_ISL_19818980 |
| hRSV/A/USA/MD-JHSOM198/2024 | EPI_ISL_19818979 |
| hRSV/A/USA/MD-JHSOM197/2024 | EPI_ISL_19818978 |
| hRSV/A/USA/MD-JHSOM196/2024 | EPI_ISL_19818977 |
| hRSV/A/USA/MD-JHSOM195/2024 | EPI_ISL_19818976 |
| hRSV/A/USA/MD-JHSOM193/2024 | EPI_ISL_19818975 |
| hRSV/A/USA/MD-JHSOM192/2024 | EPI_ISL_19818974 |
| hRSV/A/USA/MD-JHSOM190/2024 | EPI_ISL_19818973 |
| hRSV/A/USA/MD-JHSOM189/2024 | EPI_ISL_19818972 |
| hRSV/A/USA/MD-JHSOM188/2024 | EPI_ISL_19818971 |
| hRSV/A/USA/MD-JHSOM187/2024 | EPI_ISL_19818970 |
| hRSV/A/USA/MD-JHSOM186/2024 | EPI_ISL_19818969 |
| hRSV/A/USA/MD-JHSOM185/2024 | EPI_ISL_19818968 |
| hRSV/A/USA/MD-JHSOM184/2024 | EPI_ISL_19818967 |
| hRSV/A/USA/MD-JHSOM183/2024 | EPI_ISL_19818966 |
| hRSV/A/USA/MD-JHSOM182/2024 | EPI_ISL_19818965 |
| hRSV/A/USA/MD-JHSOM181/2024 | EPI_ISL_19818964 |
| hRSV/A/USA/MD-JHSOM178/2024 | EPI_ISL_19818963 |
| hRSV/A/USA/MD-JHSOM177/2024 | EPI_ISL_19818962 |
| hRSV/A/USA/MD-JHSOM176/2024 | EPI_ISL_19818961 |
| hRSV/B/USA/MD-JHSOM175/2024 | EPI_ISL_19818960 |
| hRSV/A/USA/MD-JHSOM174/2024 | EPI_ISL_19818959 |
| hRSV/A/USA/MD-JHSOM173/2024 | EPI_ISL_19818958 |
| hRSV/A/USA/MD-JHSOM172/2024 | EPI_ISL_19818957 |
| hRSV/A/USA/MD-JHSOM171/2024 | EPI_ISL_19818956 |
| hRSV/A/USA/MD-JHSOM170/2024 | EPI_ISL_19818955 |
| hRSV/A/USA/MD-JHSOM169/2024 | EPI_ISL_19818954 |
| hRSV/A/USA/MD-JHSOM168/2024 | EPI_ISL_19818953 |
| hRSV/A/USA/MD-JHSOM167/2024 | EPI_ISL_19818952 |
| hRSV/A/USA/MD-JHSOM166/2024 | EPI_ISL_19818951 |
| hRSV/A/USA/MD-JHSOM165/2024 | EPI_ISL_19818950 |
| hRSV/A/USA/MD-JHSOM161/2024 | EPI_ISL_19818949 |
| hRSV/A/USA/MD-JHSOM160/2024 | EPI_ISL_19818948 |
| hRSV/A/USA/MD-JHSOM158/2024 | EPI_ISL_19818947 |
| hRSV/A/USA/MD-JHSOM157/2024 | EPI_ISL_19818946 |
| hRSV/A/USA/MD-JHSOM156/2024 | EPI_ISL_19818945 |
| hRSV/A/USA/MD-JHSOM154/2024 | EPI_ISL_19818944 |
| hRSV/A/USA/MD-JHSOM152/2024 | EPI_ISL_19818943 |
| hRSV/A/USA/MD-JHSOM151/2024 | EPI_ISL_19818942 |
| hRSV/A/USA/MD-JHSOM150/2024 | EPI_ISL_19818941 |
| hRSV/A/USA/MD-JHSOM149/2024 | EPI_ISL_19818940 |
| hRSV/A/USA/MD-JHSOM147/2024 | EPI_ISL_19818939 |
| hRSV/A/USA/MD-JHSOM146/2024 | EPI_ISL_19818938 |
| hRSV/A/USA/MD-JHSOM142/2024 | EPI_ISL_19818937 |
| hRSV/A/USA/MD-JHSOM141/2024 | EPI_ISL_19818936 |
| hRSV/A/USA/MD-JHSOM140/2024 | EPI_ISL_19818935 |
| hRSV/A/USA/MD-JHSOM139/2024 | EPI_ISL_19818934 |
| hRSV/A/USA/MD-JHSOM138/2024 | EPI_ISL_19818933 |
| hRSV/A/USA/MD-JHSOM137/2024 | EPI_ISL_19818932 |
| hRSV/A/USA/MD-JHSOM136/2024 | EPI_ISL_19818931 |
| hRSV/A/USA/MD-JHSOM135/2024 | EPI_ISL_19818930 |
| hRSV/A/USA/MD-JHSOM134/2024 | EPI_ISL_19818929 |
| hRSV/A/USA/MD-JHSOM133/2024 | EPI_ISL_19818928 |
| hRSV/A/USA/MD-JHSOM132/2024 | EPI_ISL_19818927 |
| hRSV/A/USA/MD-JHSOM131/2024 | EPI_ISL_19818926 |
| hRSV/A/USA/MD-JHSOM130/2024 | EPI_ISL_19818925 |
| hRSV/A/USA/MD-JHSOM129/2024 | EPI_ISL_19818924 |
| hRSV/A/USA/MD-JHSOM128/2024 | EPI_ISL_19818923 |
| hRSV/A/USA/MD-JHSOM127/2024 | EPI_ISL_19818922 |
| hRSV/A/USA/MD-JHSOM126/2024 | EPI_ISL_19818921 |
| hRSV/A/USA/MD-JHSOM125/2024 | EPI_ISL_19818920 |
| hRSV/A/USA/MD-JHSOM124/2024 | EPI_ISL_19818919 |
| hRSV/A/USA/MD-JHSOM123/2024 | EPI_ISL_19818918 |
| hRSV/B/USA/MD-JHSOM122/2024 | EPI_ISL_19818917 |
| hRSV/A/USA/MD-JHSOM121/2024 | EPI_ISL_19818916 |
| hRSV/A/USA/MD-JHSOM119/2024 | EPI_ISL_19818915 |
| hRSV/A/USA/MD-JHSOM118/2024 | EPI_ISL_19818914 |
| hRSV/A/USA/MD-JHSOM117/2024 | EPI_ISL_19818913 |
| hRSV/A/USA/MD-JHSOM116/2024 | EPI_ISL_19818912 |
| hRSV/A/USA/MD-JHSOM115/2024 | EPI_ISL_19818911 |
| hRSV/A/USA/MD-JHSOM114/2024 | EPI_ISL_19818910 |
| hRSV/A/USA/MD-JHSOM113/2024 | EPI_ISL_19818909 |
| hRSV/A/USA/MD-JHSOM111/2024 | EPI_ISL_19818908 |
| hRSV/A/USA/MD-JHSOM110/2024 | EPI_ISL_19818907 |
| hRSV/A/USA/MD-JHSOM108/2024 | EPI_ISL_19818906 |
| hRSV/A/USA/MD-JHSOM107/2024 | EPI_ISL_19818905 |
| hRSV/A/USA/MD-JHSOM106/2024 | EPI_ISL_19818904 |
| hRSV/A/USA/MD-JHSOM105/2024 | EPI_ISL_19818903 |
| hRSV/A/USA/MD-JHSOM104/2024 | EPI_ISL_19818902 |
| hRSV/A/USA/MD-JHSOM103/2024 | EPI_ISL_19818901 |
| hRSV/A/USA/MD-JHSOM102/2024 | EPI_ISL_19818900 |
| hRSV/A/USA/MD-JHSOM101/2024 | EPI_ISL_19818899 |
| hRSV/A/USA/MD-JHSOM98/2024 | EPI_ISL_19818898 |
| hRSV/A/USA/MD-JHSOM96/2024 | EPI_ISL_19818897 |
| hRSV/A/USA/MD-JHSOM94/2024 | EPI_ISL_19818896 |
| hRSV/A/USA/MD-JHSOM93/2024 | EPI_ISL_19818895 |
| hRSV/A/USA/MD-JHSOM92/2024 | EPI_ISL_19818894 |
| hRSV/A/USA/MD-JHSOM91/2024 | EPI_ISL_19818893 |
| hRSV/A/USA/MD-JHSOM89/2024 | EPI_ISL_19818892 |
| hRSV/A/USA/MD-JHSOM87/2024 | EPI_ISL_19818891 |
| hRSV/A/USA/MD-JHSOM86/2024 | EPI_ISL_19818890 |
| hRSV/A/USA/MD-JHSOM84/2024 | EPI_ISL_19818889 |
| hRSV/A/USA/MD-JHSOM83/2024 | EPI_ISL_19818888 |
| hRSV/A/USA/MD-JHSOM82/2024 | EPI_ISL_19818887 |
| hRSV/A/USA/MD-JHSOM81/2024 | EPI_ISL_19818886 |
| hRSV/A/USA/MD-JHSOM80/2024 | EPI_ISL_19818885 |
| hRSV/B/USA/MD-JHSOM79/2024 | EPI_ISL_19818884 |
| hRSV/A/USA/MD-JHSOM78/2024 | EPI_ISL_19818883 |
| hRSV/B/USA/MD-JHSOM77/2024 | EPI_ISL_19818882 |
| hRSV/A/USA/MD-JHSOM76/2024 | EPI_ISL_19818881 |
| hRSV/A/USA/MD-JHSOM75/2024 | EPI_ISL_19818880 |
| hRSV/A/USA/MD-JHSOM74/2024 | EPI_ISL_19818879 |
| hRSV/A/USA/MD-JHSOM73/2024 | EPI_ISL_19818878 |
| hRSV/A/USA/MD-JHSOM72/2024 | EPI_ISL_19818877 |
| hRSV/A/USA/MD-JHSOM71/2024 | EPI_ISL_19818876 |
| hRSV/A/USA/MD-JHSOM69/2024 | EPI_ISL_19818875 |
| hRSV/A/USA/MD-JHSOM68/2024 | EPI_ISL_19818874 |
| hRSV/A/USA/MD-JHSOM67/2024 | EPI_ISL_19818873 |
| hRSV/A/USA/MD-JHSOM65/2024 | EPI_ISL_19818872 |
| hRSV/A/USA/MD-JHSOM64/2024 | EPI_ISL_19818871 |
| hRSV/A/USA/MD-JHSOM63/2024 | EPI_ISL_19818870 |
| hRSV/A/USA/MD-JHSOM62/2024 | EPI_ISL_19818869 |
| hRSV/A/USA/MD-JHSOM61/2024 | EPI_ISL_19818868 |
| hRSV/A/USA/MD-JHSOM59/2024 | EPI_ISL_19818867 |
| hRSV/A/USA/MD-JHSOM57/2024 | EPI_ISL_19818866 |
| hRSV/A/USA/MD-JHSOM56/2024 | EPI_ISL_19818865 |
| hRSV/A/USA/MD-JHSOM55/2024 | EPI_ISL_19818864 |
| hRSV/A/USA/MD-JHSOM54/2024 | EPI_ISL_19818863 |
| hRSV/A/USA/MD-JHSOM53/2024 | EPI_ISL_19818862 |
| hRSV/B/USA/MD-JHSOM52/2024 | EPI_ISL_19818861 |
| hRSV/A/USA/MD-JHSOM51/2024 | EPI_ISL_19818860 |
| hRSV/A/USA/MD-JHSOM50/2024 | EPI_ISL_19818859 |
| hRSV/A/USA/MD-JHSOM49/2024 | EPI_ISL_19818858 |
| hRSV/B/USA/MD-JHSOM47/2024 | EPI_ISL_19818857 |
| hRSV/A/USA/MD-JHSOM46/2024 | EPI_ISL_19818856 |
| hRSV/A/USA/MD-JHSOM45/2024 | EPI_ISL_19818855 |
| hRSV/A/USA/MD-JHSOM44/2024 | EPI_ISL_19818854 |
| hRSV/B/USA/MD-JHSOM43/2024 | EPI_ISL_19818853 |
