# Supplementary material for: Whole-genome sequence characterization of respiratory syncytial virus in the Johns Hopkins Health System during the 2024–2025 respiratory season
Source: Microbiol Spectr. 2025 Oct 7;13(11):e02065-25. doi: 10.1128/spectrum.02065-25 (PMC12584621; doi:10.1128/spectrum.02065-25)
Supplement: Table S2 — Primers and probes used for RSV amplification and detection. [file spectrum.02065-25-s0002.docx]

Supplementary Table S2. Primers and probe used for RT-qPCR and primers for two-step RT-PCR

| **RT-qPCR** | | |
| --- | --- | --- |
| **Primer sequence (5’-3’)** | | **Final conc (nM)** |
| F: GGCAAATATGGAAACATACGTGAA | | 400 |
| R: TCTTTTTCTAGGACATTGTAYTGAACAG | | 400 |
| P: CTGTGTATGTGGAGCCTTCGTGAAGCT (5’ end was labeled with FAM and 3’end was quenched with Iowa Black FQ (Integrated DNA Technologies)) | | 200 |
| **Two-step RT-PCR** | | |
| **Pool 1 or 2** | **Primer sequence (5'–3')** | **Final conc (nM)** |
| Pool 1 | Amp 1F: AAAAATGCGTACWACAAACTTGC | 400 |
|  | Amp 1R: TTGATTGMAAAWCGTGTAGCT | 400 |
|  | Amp 3F: TGATGCATCAATATCTCAAGTC | 400 |
|  | Amp 3R1: AGGACTTTCTTTATACTAGCTG | 200 |
|  | Amp 3R2: AGGACTTTTTTGATACTGGCTG | 200 |
| Pool 2 | Amp 2F: GCCACARAGTCAATTYATAGTAG | 200 |
|  | Amp 2R: TGTRACTGGTGTGYTTYTGG | 200 |
|  | Amp 4F: CTCAAGCAGATTATTTGYTAGCA | 400 |
|  | Amp 4R1: TTGAATACAATGTTAGTGTGTAGC | 400 |
|  | Amp 4R2: GTTGTAAATGCACATGTGTGATTG | 400 |

**Abbreviations:** F, forward primer; R, reverse primer; P, probe; Amp, amplicon; RT, reverse transcription; qPCR, quantitative polymerase chain reaction
