# Supplementary material for: Whole-genome sequence characterization of respiratory syncytial virus in the Johns Hopkins Health System during the 2024–2025 respiratory season
Source: Microbiol Spectr. 2025 Oct 7;13(11):e02065-25. doi: 10.1128/spectrum.02065-25 (PMC12584621; doi:10.1128/spectrum.02065-25)
Supplement: Table S3 — Reference genomes used for the phylogenetic analysis. [file spectrum.02065-25-s0003.docx]

Supplementary Table S3. Reference genomes from GenBank used for the phylogenetic analysis

| **RSV-A Reference** |
| --- |
| JF920059.2_hRSV-A/USA/2023 |
| LC862068.1_Human_respiratory_syncytial_virus_A_HRSV/A/Japan/21U125iso/2021_RNA_complete_sequence |
| PQ618067.1_Human_respiratory_syncytial_virus_A_isolate_hRSV/A/Panama/ICGES-196/2018_complete_genome |
| ON237252.1_Human_respiratory_syncytial_virus_A_isolate_HRSV/A/Argentina/BA-HNRG-125/2015_complete_genome |
| JX015488.1_hRSV-A/Netherlands/2006 |
| KP258715.1_hRSV-A/USA/1988 |
| PP974117.1_hRSV-A/China/2015 |
| OK649646.1_hRSV-A/Brazil/2010 |
| OR287939.1_hRSV-A/USA/2020 |
| PP495893.1_hRSV-A/USA/2019 |
| MF001047.2_hRSV-A/USA/2010 |
| PP748708.1_hRSV-A/USA/2019 |
| OM857175.1_hRSV-A/Australia/2021 |
| PP376515.1_hRSV-A/Netherlands/2021 |
| OM857231.1_hRSV-A/Australia/2020 |
| OM857173.1_hRSV-A/Australia/2020 |
| OY757638.1_hRSV-A/Australia/2023 |
| PP352384.1_hRSV-A/USA/2022 |
| OR975306.1_hRSV-A/USA/2023 |
| PQ638722.1_hRSV-A/USA/2023 |
| PQ763086.1_hRSV-A/France/2024 |
| PP934452.1_hRSV-A/USA/2024 |
| PQ618039.1_hRSV-A/Panama/2023 |
| PP847379.1_hRSV-A/USA/2024 |
| PP973752.1_hRSV-A/USA/2024 |
| PP495957.1_hRSV-A/USA/2021 |
| OR872587.1_hRSV-A/USA/2022 |
| PQ638731.1_hRSV-A/USA/2021 |
| ON237253.1_hRSV-A/Argentina/2015 |
| MZ515797.1_hRSV-A/UK/2020 |
| PP974151.1_hRSV-A/China/2021 |
| OR666551.1_hRSV-A/China/2020 |
| MT422271.1_hRSV-A/Russia/2019 |
| PP969953.1_hRSV-A/Ireland/2022 |
| PQ348824.1_hRSV-A/UK/2021 |
| PV080993.1_hRSV-A/USA/2025 |
| PV080950.1_hRSV-A/USA/2025 |
| PP084060.1_hRSV-A/USA/2023 |
| OR522494.1_hRSV-A/USA/2022 |
| PQ348892.1_hRSV-A/UK/2022 |
| PP342430.1_hRSV-A/USA/2023 |
| PQ348854.1_hRSV-A/UK/2021 |
| PP504630.1_hRSV-A/USA/2023 |
| PP910752.1_hRSV-A/USA/2023 |
| PP386344.1_hRSV-A/USA/2023 |
| PP377526.1_hRSV-A/Netherlands/2020 |
| PP377588.1_hRSV-A/Netherlands/2019 |
| PP376503.1_hRSV-A/Netherlands/2019 |
| PP970002.1_hRSV-A/Ireland/2023 |
| PQ349024.1_hRSV-A/UK/2023 |
| PQ737350.1_hRSV-A/France/2023 |
| OR795471.1_hRSV-A/Germany2022 |
| PP352318.1_hRSV-A/USA/2020 |
| PQ788251.1_hRSV-A/USA/2024 |
| hRSV/A/Mexico/MEX_InDRE_486/2024\|EPI_ISL_19814382\|2024-12-19 |
| hRSV/A/Mexico/MEX_InDRE_499/2025\|EPI_ISL_19814384\|2025-01-07 |
| hRSV/A/Ukraine/RLDHA-3873/2025\|EPI_ISL_19862072\|2025-03-12 |
| hRSV/A/Ukraine/RLDHA-2905/2024\|EPI_ISL_19862074\|2024-12-22 |
| hRSV/A/Spain/GA-CHUVI-36386262/2025\|EPI_ISL_19863102\|2025-01-27 |
| hRSV/A/Spain/GA-CHUVI-52456380/2025\|EPI_ISL_19863105\|2025-01-21 |
| hRSV/A/Norway/01573/2025\|EPI_ISL_19863335\|2025-01-27 |
| hRSV/A/Bangladesh/IEDCR082647/2024\|EPI_ISL_19863363\|2024-06-11 |
| hRSV/A/Bangladesh/IEDCR152024/2024\|EPI_ISL_19863362\|2024-11-15 |
| hRSV/A/Italy/LIG-02-4/2025\|EPI_ISL_19866260\|2025-04-01 |
| hRSV/A/Italy/LIG-21-2/2025\|EPI_ISL_19866268\|2025-02-21 |
| hRSV/A/Nepal/NPHL0010/2024\|EPI_ISL_19871555\|2024-12-30 |
| hRSV/A/Nepal/NPHL0016/2024\|EPI_ISL_19871557\|2024-12-31 |
| hRSV/A/South_Africa/PATH-CERI-C067419/2025\|EPI_ISL_19871844\|2025-03-19 |
| hRSV/A/South_Africa/PATH-CERI-C067416/2025\|EPI_ISL_19871842\|2025-03-13 |
| hRSV/A/France/ARA-HCL025005855401/2025\|EPI_ISL_19876265\|2025-01-10 |
| hRSV/A/Peru/LIM-UPCH-4926/2025\|EPI_ISL_19873136\|2025-02-24 |
| **RSV-B Reference** |
| OM857393.1_hRSV-B/Australia/2020 |
| OR795249.1_hRSV-B/Germany/2021 |
| OR915844.1_hRSV-B/USA/2023 |
| OP890343.1_hRSV-B/USA/2022 |
| OR795286.1_hRSV-B/Germany/2022 |
| PQ492129.2_hRSV-B/Argentina/2024 |
| PV081087.1_hRSV-B/USA/2025 |
| PQ492117.2_hRSV-B/Argentina/2024 |
| PP748831.1_hRSV-B/USA/2024 |
| PP934503.1_hRSV-B/USA/2024 |
| PV016967.1_hRSV-B/USA/2024 |
| PV016977.1_hRSV-B/USA/2024 |
| PQ849401.1_hRSV-B/USA/2024 |
| OR666606.1_hRSV-B/China/2020 |
| OM857385.1_hRSV-B/Australia/2021 |
| PQ638761.1_hRSV-B/USA/2023 |
