# Supplementary material for: Whole-genome sequence characterization of respiratory syncytial virus in the Johns Hopkins Health System during the 2024–2025 respiratory season
Source: Microbiol Spectr. 2025 Oct 7;13(11):e02065-25. doi: 10.1128/spectrum.02065-25 (PMC12584621; doi:10.1128/spectrum.02065-25)
Supplement: Table S4 — Amino acid substitutions identified in RSV sequences. [file spectrum.02065-25-s0004.docx]

Supplementary Table S4. Whole-genome amino acid substitutions and their frequencies among RSV-A strains.

| Gene | Substitution | Frequency |
| --- | --- | --- |
| NS-2 | R50K | 77.78% |
| N | V352A | 100.0% |
| P | L55P | 100.0% |
| M | M43I | 86.62% |
| SH | I38S | 71.11% |
| G | \| A57T \| \| --- \| \| P71L \| \| H90Y \| \| S100N \| \| L101F \| \| G106E \| \| P120L \| \| S121I \| \| I134K \| \| T137K \| \| L142S \| \| P143S \| \| P217S \| \| G224E \| \| G224V \| \| P230H \| \| N242T \| \| S243I \| \| K262E \| \| I265L \| \| D284G \| \| S294P \| \| G296S \| \| S299N \| \| Y304H \| \| T319A \| \| T319I \| \| T320A \| | \| 11.04% \| \| --- \| \| 99.69% \| \| 99.37% \| \| 11.08% \| \| 98.75% \| \| 71.34% \| \| 68.89% \| \| 68.89% \| \| 100.00% \| \| 11.64% \| \| 84.76% \| \| 68.79% \| \| 68.47% \| \| 88.92% \| \| 11.08% \| \| 10.69% \| \| 68.79% \| \| 99.37% \| \| 99.37% \| \| 99.68% \| \| 100.00% \| \| 11.08% \| \| 68.79% \| \| 69.52% \| \| 94.30% \| \| 69.45% \| \| 19.94% \| \| 91.43% \| |
| F | \| A10T \| \| --- \| \| T12I \| \| T13A \| \| C21W \| \| I59V \| \| S99N \| \| A103T \| \| T122A \| \| N124T \| \| V127I \| \| K272N \| \| S276N \| \| S377N \| | \| 7.23% \| \| --- \| \| 5.99% \| \| 1.89% \| \| 2.52% \| \| 7.23% \| \| 11.67% \| \| 8.81% \| \| 8.81% \| \| 7.23% \| \| 11.04% \| \| 4.44% \| \| 2.20% \| \| 2.52% \| |
| M2-1 | \| N174S \| \| --- \| \| S176P \| \| T180A \| | \| 12.15% \| \| --- \| \| 100.00% \| \| 12.11% \| |
| M2-2 | \| T1M \| \| --- \| \| Y26C \| \| S46N \| | \| 12.37% \| \| --- \| \| 90.94% \| \| 100.00% \| |
| L | \| S100L \| \| --- \| \| N143D \| \| P171L \| \| T179S \| \| R256K \| \| Y598H \| \| M1225I \| \| L1438Q \| \| I1653V \| \| K1661N \| \| H1690Y \| \| H1707Q \| \| N1719Y \| \| N1723S \| \| N1724D \| \| E1725G \| \| G1731D \| \| Y2163N \| | \| 11.15% \| \| --- \| \| 78.87% \| \| 100.00% \| \| 79.65% \| \| 100.00% \| \| 100.00% \| \| 81.25% \| \| 100.00% \| \| 88.19% \| \| 86.81% \| \| 65.49% \| \| 65.07% \| \| 64.83% \| \| 91.84% \| \| 64.38% \| \| 100.00% \| \| 100.00% \| \| 65.42% \| |
