# Supplementary material for: Whole-genome sequence characterization of respiratory syncytial virus in the Johns Hopkins Health System during the 2024–2025 respiratory season
Source: Microbiol Spectr. 2025 Oct 7;13(11):e02065-25. doi: 10.1128/spectrum.02065-25 (PMC12584621; doi:10.1128/spectrum.02065-25)
Supplement: Table S5 — Calculated odds ratios used for the forest plot analyses. [file spectrum.02065-25-s0005.docx]

Supplementary Table S5. Odds ratio and the associated 95% confidence interval. Significant association between clinical outcomes and characteristics was bolded.

|  | Odds ratio (95% Confidence Interval) (p-value) | | |
| --- | --- | --- | --- |
|  | Admitted | Supplemental Oxygen | ICU-level care |
| Male | 1 (Reference) | | |
| Female | 1.10 (0.65 - 1.86) (0.73) | 0.74 (0.41 - 1.34) (0.33) | 0.46 (0.15 - 1.36) (0.16) |
| Subtype |  | | |
| A | 1 (Reference) | | |
| B | 1.14 (0.36 - 3.60) (0.83) | 1.10 (0.31 - 3.96) (0.88) | 1.27 (0.16 - 10.20) (0.82) |
| Patient age |  | | |
| 0 - 1 | 1 (Reference) | | |
| 1 - 5 | 0.65 (0.34 - 1.24) (0.33) | 0.65 (0.33 - 1.29) (0.22) | **0.26 (0.07 - 0.90) (0.03)** |
| 6 - 17 | 1.62 (0.66 - 3.99) (0.29) | 1.16 (0.43 - 3.10) (0.77) | 1.19 (0.29 - 4.88) (0.81) |
| 18 - 59 | 2.49 (0.89 - 6.96) (0.08) | 0.76 (0.20 - 2.88) (0.68) | 0.62 (0.07 - 5.38) (0.67) |
| >= 60 | **11.21 (1.10 - 113.98) (0.04)** | 1.43 (0.14 - 14.62) (0.76) | 0 |
| Comorbidities |  | | |
| Asthma | **3.25 (1.31 - 8.08) (0.01)** | **4.62 (1.84 – 11.60) (0.001)** | 2.45 (0.51 – 11.63) (0.26) |
| Atrial fibrillation | 4.03 (0.56 - 29.15) (0.17) | 1.79 (0.18 - 17.59) (0.62) | 0 |
| Cancer | **2.75 (1.40 - 5.37) (0.003)** | 1.60 (0.74 - 3.45) (0.24) | 0.97 (0.21 - 4.44) (0.97) |
| Cerebrovascular disease | **5.52 (1.21 - 25.28) (0.03)** | 4.19 (0.91 - 19.27) (0.07) | 3.75 (0.42 - 33.30) (0.24) |
| Coronary artery disease | **4.33 (1.56 - 12.01) (0.005)** | 1.84 (0.57 - 5.95) (0.31) | 1.46 (0.18 - 11.83) (0.72) |
| Diabetes | **10.13 (2.55 - 40.33) (0.001)** | 2.37 (0.59 - 9.46) (0.22) | 2.48 (0.29 - 20.93) (0.41) |
| Heart failure | **12.87 (2.54 - 65.30) (0.002)** | **5.69 (1.38 - 23.53) (0.02)** | 3.20 (0.37 - 27.86) (0.29) |
| Hypertension | **8.14 (3.39 - 19.59) (0.000003)** | **2.97 (1.20 - 7.34) (0.02)** | 3.57 (0.93 - 13.64) (0.06) |
| Immunosuppression | **8.05 (4.00 - 16.20) (0.000000005)** | **2.21 (1.03 - 4.74) (0.04)** | **3.96 (1.28 - 12.23) (0.02)** |
| Kidney disease | **10.15 (3.40 - 30.35) (0.00003)** | **4.63 (1.64 - 13.05) (0.004)** | 3.37 (0.69 - 16.41) (0.13) |
| Non-asthmatic lung disease | 1.85 (0.97 - 3.51) (0.06) | 1.95 (0.98 - 3.89) (0.06) | 0.73 (0.16 - 3.32) (0.68) |
| Smoker | **10.56 (2.00 - 55.66) (0.005)** | 2.18 (0.41 - 11.55) (0.36) | 3.75 (0.42 - 33.30) (0.24) |
| Emergency department visit | 0.54 (0.18 - 1.61) (0.27) | 1.33 (0.29 - 6.02) (0.71) | 0.69 (0.08 - 5.57) (0.72) |
